# Raman microscopy tracks maturity of melanin intermediates in *Botrytis cinerea*, a plant pathogen†

**DOI:** 10.1039/d2ra06439a

**Published:** 2023-01-06

**Authors:** Victor V. Volkov, Ayesha Sadaf, Carole C. Perry

**Affiliations:** a Interdisciplinary Biomedical Research Centre, School of Science and Technology, Nottingham Trent University Nottingham NG11 8NS UK +44 (0)115 8486695 carole.perry@ntu.ac.uk

## Abstract

We use Raman microscopy to describe the structure and chemical composition of both conidiophore and hyphae of *Botrytis cinerea*, a common plant pathogen. To interpret experimental data, we use density functional theory (DFT) to compute Raman tensors specific to an important fungal glycopeptide, a segment of α-chitin, and several naphthalene-based precursors of increasing complexity, which we propose play a role in the melanin synthesis pathway. Using spectral interpretations based on quantum chemical validation, we review microscopy images reconstructed for specific Raman activities and describe differences in distributions of structural components, photo-protective secondary naphthalene-based pigments, and proteins in both spores and hyphal filaments. Comparison of our results with literature data on other fungi suggests an example of convergent evolution expressed at the level of secondary metabolites specific to plant pathogenic fungi. Our results indicate that pre-resonant Raman monitoring of melanin precursors may help assessment of local *Botrytis* population biology to aid agricultural production.

## Abstract 2

Raman monitoring of melanin precursors may help assessment of local pathogenic *Botrytis cinerea* populations and support agricultural production.

## Graphical abstract

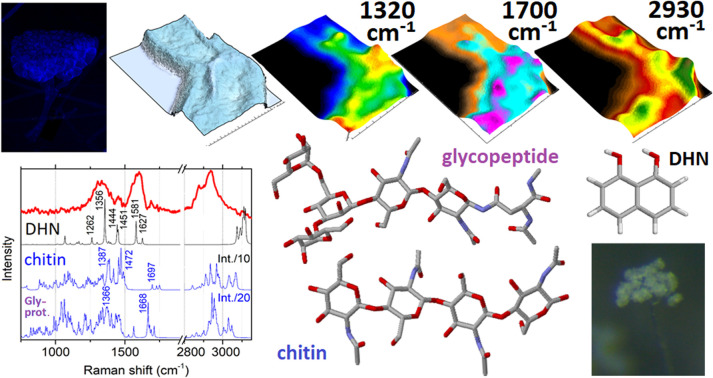

## 1. Introduction

Experimental Raman spectroscopy^1–3^ and Raman imaging microscopy^4,5^ have been used to describe chemical processes in biological tissues that relate to the structure of various living species, including fungi. For example, Raman spectroscopy helped to characterize fungal cell wall components and metabolite activities.^6,7^ However, the results of pioneering Raman studies in fungi indicated that assignment of vibrational bands in such systems may be difficult: particularly as, at the time, the level of quantum chemical theory could not help understand the complex nature of the observed Raman transitions.^8^ Additionally, low sensitivity of detection and strong fluorescence backgrounds were seen^5^ as serious obstacles to the application of Raman diagnostics to biological tissues in general, and to fungal specimens, in particular.

In recent years, significant improvement of Raman signal detection has become possible following the introduction of surface-enhanced Raman scattering (SERS)^9^ and demonstration of multiphoton nonlinear coherent anti-stokes Raman scattering (CARS) stimulation.^10^ For SERS, the Raman cross section of molecules adsorbed onto silver or gold nanoparticles may increase by many orders of magnitude.^11^ For example, using Klarite SERS substrates, traces of *Aspergillus nidulans* fungal hyphae excretions have been detected.^12^ This approach has been helpful in reporting Raman signatures of fungal pathogens including *Trichophyton rubrum*, *Candida krusei*, *Scopulariopsis brumptii*, and *Aspergillus flavus*.^13^

SERS spectroscopy is also being used for identification of a ‘type’ of molecular species, however this approach has some limitations: SERS spectra might not be easy to interpret, may not be easy for quantitative analysis. To properly understand the SERS spectrum, many factors must be taken into consideration. First, factors affecting enhancement of the Raman signal which include the precise electromagnetic interactions and chemical mechanism leading to signal enhancement is required.^14^ Second, since sensitive to distance (the strongest effect is observed within 10 nm of the active substrate), a response signal should include superposition of various SERS signals under different nonlinear enhancements. Third, since variances of the electronic properties of active substrates (space and time-dependent) may induce additional heterogeneity, sampling time scales should be accounted carefully: for example, blinking of gold colloids was reported on the timescale of milliseconds.^15,16^ Further, preparation and characterization of ultrafast pulses has been successfully employed to stimulate coherent Raman responses of significantly higher intensity (due to phase matching and intense fields) than usually obtained for spontaneous signals. As an example, the approach was used to describe the distribution of cytochromes in hyphal tip cells of *Schizophyllum commune*.^17^ Similarly, CARS has been effective in identifying the presence of glucans in the cell wall, as well as cytoplasmic contents- and nucleotides, proteins, polysaccharides, and lipids in *Aspergillus nidulans* spores.^18^ Quantitative analysis is possible but requires modelling over various possible orientational distributions. In this respect, CARS is very important for demonstrations but currently challenging for quantitative analysis.

Progress in the technology of photodetectors has “revitalized” conventional Raman microscopy. For example, the approach has enabled the visualization of the Raman signatures of cell wall components such as α-glucan, β-glucan and mannan in a single living fission yeast vegetative cell and in a spore of *Schizosaccharomyces pombe*.^19^ Also, facilitated by electronic pre-resonance, a recent Raman study reported the relative abundance of cytochromes b and c in their redox states (reduced *vs.* oxidized form) among three different representative compartments of *Aspergillus nidulans* hyphae.^20^ Additionally, a Raman difference spectroscopy study, using a near infrared laser at 785 nm, suggested a promising alternative to sample Raman spectra in hyphal specimens: the authors discussed synthesis of DHN melanin in *Aspergillus fumigatus* and other saprotrophic fungi.^21^

Although a few previous studies have demonstrated the ability of Raman microscopy to explore the signature of chemical components in fungal filaments, there are still major issues with the technique which need to be overcome for more widespread application. Firstly, fungal structures may not provide intense Raman signals due to a lack of chromophores with pre-resonance enhancement. At the same time, fungal filaments may not be structurally and chemically stable to tolerate fluence increase to stimulate suitable signals.^22^ A recent successful Raman microscopy report on the branched mycelium of *Colletotrichum camelliae Massee* (contemporary name *Colletotrichum coccodes*), a pathogen that infects potato, onion, tomato, and many other plants^23^ ascribed Raman signatures mainly to chitin and suggested a description for distribution of this molecular component in space. Since the study of fungi as both pathogens and as vegan replacements for fabrics is gaining momentum and more studies are required to correlate composition and structure of other fungal hyphae. In this case we study the ‘grey mould’ *Botrytis cinerea*.

The genus *Botrytis* belongs to the family of necrotrophic plant pathogens,^24,25^ comprises at least 22 species,^26,27^ and infects more than 1400 different species of plants of commercial value resulting in large losses.^28,29^ The life cycle of *Botrytis cinerea* includes mycelia to vegetate, conidia (asexual spores) for dispersal, and sclerotia, a compact mycelium for survival. Conidia are typical in summer, while in the winter sclerotia, the melanized resting bodies may germinate asexually or sexually in the following spring.^25,30^*Botrytis cinerea* is well-known for its light sensitivity and has been studied since the end of the XIX century.^31^ This fungus is known for a sophisticated system of, at least, eleven photoreceptors responsive to different UV, blue, green, red and far-red light that trigger defensive biochemistry, morphogenesis, positive and negative photo-tropisms.^32^ Genetic studies suggest that more than 40 secondary metabolites are to be expected in *B. cinerea* micro-structures.^33^ According to their chemical structures most are expected to absorb in the ultraviolet spectral range. Consistently, mycelial filaments of *B. cinerea* appear colorless or white.^32^ The conidiophores and conidia of the species acquire a greyish coloration, depending on maturity with residual or systematic deposition of 1,8-dihydroxynaphthalene (DHN)-melanin^34^ providing the characteristic color of the grey mold infection caused by these species.^35,36^

As plant pathogens damage crops,^28,29^*Botrytis* sp. are important to study. Keeping track of the biology of local populations of the species in respect to the annual agricultural cycle is necessary to balance production and natural coexistence. In this respect, effective identification of pigment-precursors suitable as indictors of growth stage may become particularly valuable because this would instruct which enzyme group may become an effective target for reduction of the pathogen to shift the balance within the local agricultural eco-community. Beside the practical purpose, understanding the physiological cascade of maturity precursors in various group of fungi may be helpful to recapitulate their evolutionary past “decisions”, when close relatives had to become different in biochemistry to compete for resources and nutrient supplies. To answer both, practical and fundamental needs we need to search for a fast and relatively cheap diagnostic to assess the physiology of plant pathogens.

**Fig. 1:**
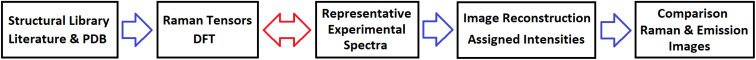
Flow chart of the analytical approach.

In this contribution, motivated by prior studies on Raman spectra of filamentous fungi,^22,23^ we used Raman microscopy operating under low power operating conditions to explore the chemistry of both the conidiophore and spore of the plant pathogen, *Botrytis cinerea*. Fig. 1 presents a flow chart of the analytical approach presented. Supported by quantum chemistry calculations we review the assignment of Raman activity specific to this fungus. We provide information on the spatial distribution of structural components, photo-protective secondary naphthalene-based pigments, and proteins to gain an understanding of the biomolecule framework and metabolism in *B. cinerea*. Comparing our results to those published for plant pathogens of different fungal classes, we propose an evolutionary convergence of secondary photo-protective precursors specific to a large group of organisms.

## 2. Materials and methods

The mould, *Botrytis cinerea* was procured from the National Collection of Pathogenic Fungi (NCPF No. 7160). The strain was maintained on Potato Dextrose Agar (PDA) slants at 4 °C and was sub-cultured every 15 days. Hyphal filaments and spores were collected from a fresh culture of the mould grown on PDA at 25 °C in the dark for 48 h. For experimental studies, we gave preference to conidiophores and filaments of the organism at the later stage of maturity, when the structures acquire greyish coloration and some spores detach readily for dispersal. The selected timing is to confirm molecular content and structural properties specific to an organism at a later stage of maturity.

Raman spectral studies were conducted using a DXR Thermo Fisher Scientific microscopy station, Madison Wisconsin, equipped with 10×, 50× and 100× objectives which was also used for bright field microscope image collection of the areas assessed by spectroscopy. Raman measurements were made using a 100× objective (numerical aperture 0.8), and 532 nm excitation radiation of 1 mW. The optimum power of the field to be used was determined by periodic focusing of radiation of different powers to confirm that filament microstructures and spores would not move (due to thermal effects) and would not demonstrate spectral signatures of degradation. For the scans reported in the article, we repeated detection of spectra at every sample point three times (each of 20 seconds accumulation) to confirm reproducibility, the absence of spurious artifacts and generate better signal to noise ratio upon averaging. To detect unambiguous spectra (free from signals of substrates) Raman spectra were measured from samples deposited on an unprotected gold mirror PFSQ05-03-M03 from Thorlabs Ltd., without any cover. All measurements were conducted at room temperature (24 °C). The spectral resolution in Raman experiments was 2 cm^−1^ according to the instrumental limit by choice of a 25-micron confocal pinhole. Raman responses at different sites of microstructures were sampled with a spatial resolution of 1 micron in both directions in the imaging plane. Spectra sampled at different sites, *i*, were fitted to extract the intensity of Raman activities, *A*_*ω*,*i*_, at frequencies of interest, *ω*, which were used in reconstructions of Raman microscopy images (RAM).^37^ Reconstructions were performed according to 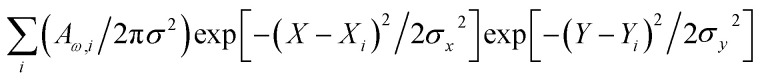, where two-dimensional Gaussian source functions were summed over all the defined sites *i*. Setting *σ*_*x*_^2^ = *σ*_*y*_^2^ = 0.5 μm^2^ provided the spatial full width of the source function. *X*_*i*_ and *Y*_*i*_ describe the position of the projection of the site *i* into the image plane. *X* and *Y* variables are sample distances from the site *i* in terms of the dimensions of detector pixels or displacements of a pinhole.

**Fig. 2:**
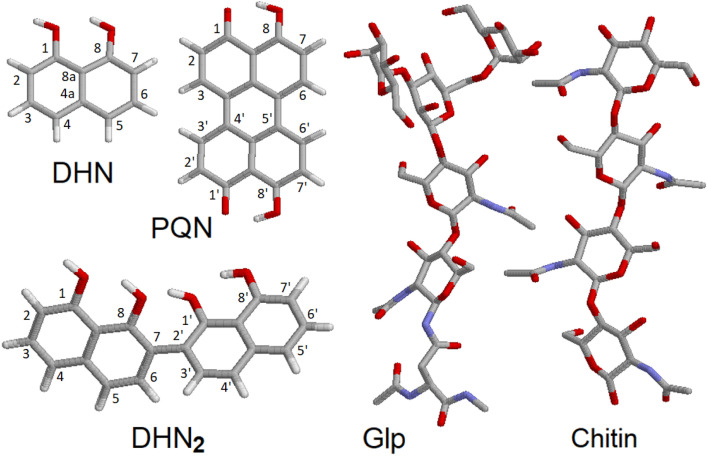
Molecular model structures. 1,8-Dihydroxynaphthalene (DHN), DHN-2-2′-dimer (DHN_2_), perylenequinone (PQN), glycopeptide (Glp), α-chitin segment.

To discuss the experimental results Raman tensors for several representative model molecular systems were calculated. These included: a glycopeptide (Glp) taken from the X-ray structure reported in 5no7.pdb for a monooxygenase of *Pycnoporus coccineus*;^38^ a segment of α-chitin; a range of precursors involved in fungal melanin synthesis, including 1,8-dihydroxynaphthalene (DHN), DHN-2-2′-dimer (DHN_2_) and perylenequinone (PQN),^39–41^ see the structures in Fig. 2. Optimisations and normal mode analysis were performed using the 6-31++g(d,p) basis set and the restricted B3LYP^42,43^ functional within the Gaussian 09 program package.^44^ Raman intensities of the normal modes were calculated for the optimized structures. A scaling factor of 0.97 was used to map DFT frequencies in the spectral range of interest. To plot Raman spectral dispersions according to DFT predictions, convolutions with Lorentzian line-shape with full width at half maximum of 8 cm^−1^ were used. To account for the proximity of the experimental excitation wavelength to naphthalene-based chromophores electronic resonances, we compute pre-resonant Raman intensities performing frequency-dependent (dynamic) coupled-perturbed Hartree–Fock equations, which are specific to the incident light frequency for the electromagnetic field perturbation: The Gaussian 09 program option cphf=rdfreq^44^ was used. For DHN and DHN_2_, the electromagnetic perturbations wavelength was selected to be at 530 nm, as in experiment. In the case of PQN, theory predicts the HOMO–LUMO transition to be at 523 nm. Because of this, to compute pre-resonance Raman intensities, the electromagnetic perturbation wavelength was set at 570 nm.

To complement the Raman studies, confocal imaging of spores and filaments treated with a fluorescent stain for chitin was conducted. The mycelium was carefully placed on a clean slide and stained with fluorochrome calcofluor white stain solution (10 μl) (Sigma-Aldrich, USA) followed by the addition of 1 M NaOH (10 μl) for better solubility of the stain. The sample was then incubated for 10–15 min at room temperature and visualised under a confocal microscope (Leica SP5 Model, Wetzlar, Germany) under 20× and 63× objectives at an excitation and emission wavelength of 380 and 475 nm respectively.

## 3. Results and discussion

**Fig. 3:**
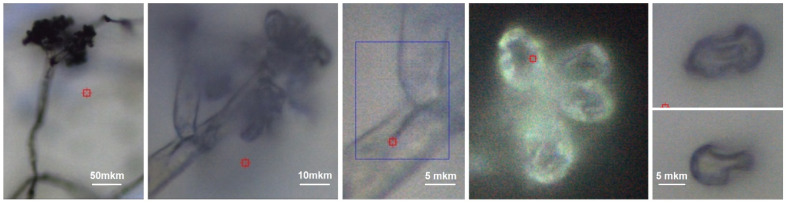
Bright field microscopy of a selected conidiophore of *Botrytis cinerea* using 10×, 50× and 100× objectives (from left to right). Blue box in the image sampled with 100× microscope objective and indicates the filament area selected for confocal Raman microscopy. Images on the right demonstrate not trivial shapes of spores. In each image, the red box indicates the center of the area imaged by the objective.

**Fig. 4:**
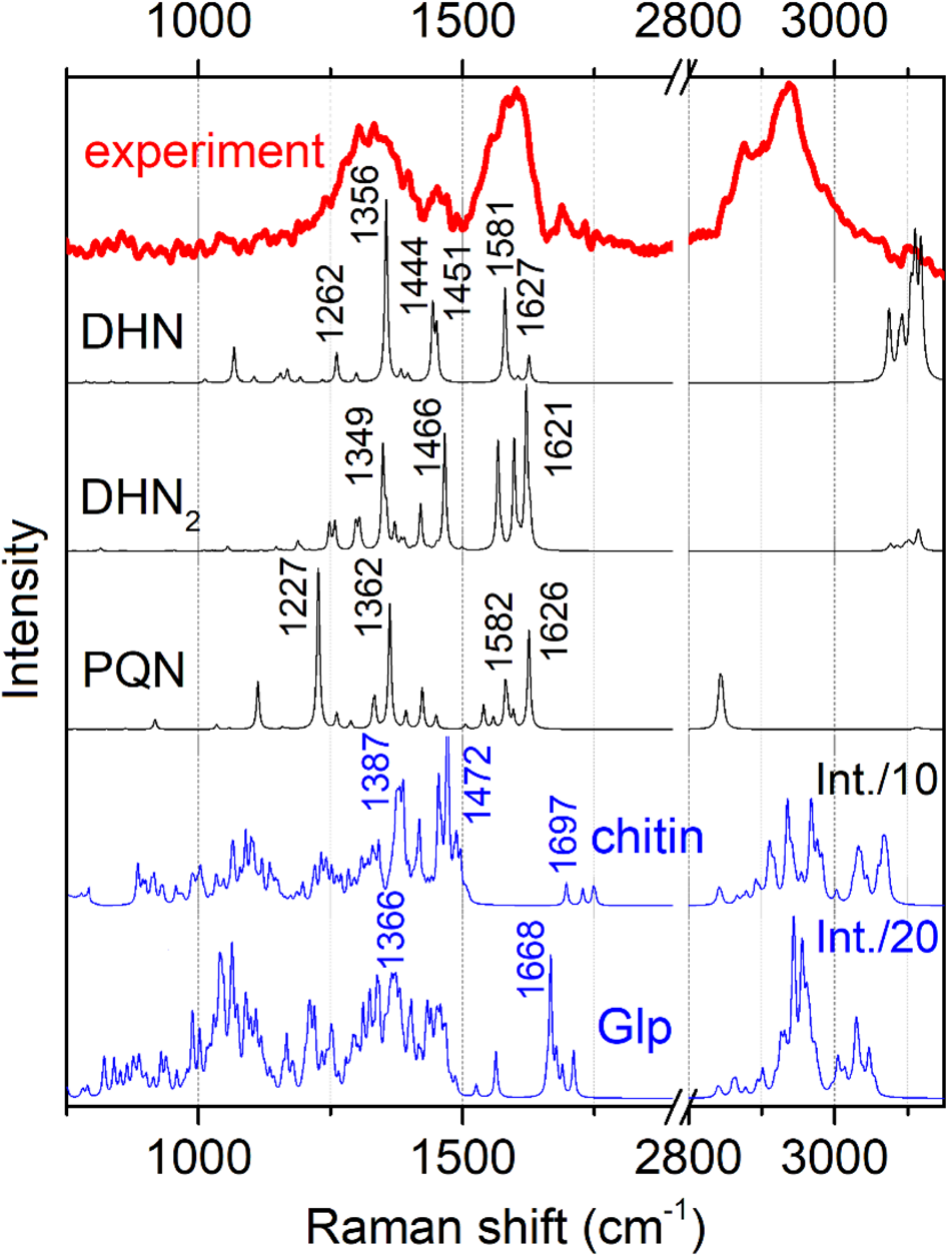
Experimental representative Raman spectrum (top) and computed Raman spectra for the model chemical structures, as shown in Fig. 2. To account for naphthalene-based chromophores, we present pre-resonant spectra: see Materials and methods.

**Fig. 5:**
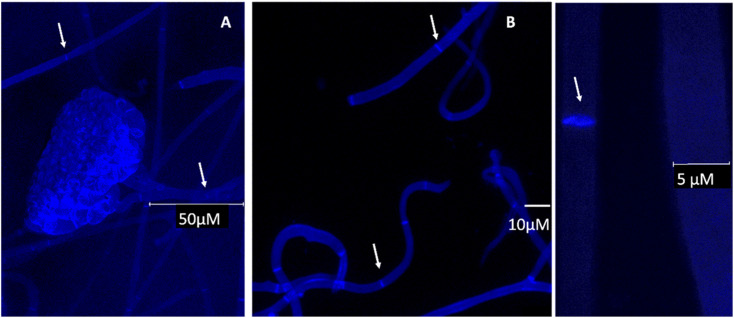
Confocal microscopy images of selected *conidiophore* and hyphae of *B. cinerea* stained with calcofluor white indicating the presence of chitin and true hyphal structures (arrows pointing towards septum). A and C under 63× and B under 20× objective respectively, scale bars as shown in the individual images.

Fig. 3 shows conidiophore, a hyphal branched stem under higher magnification and spores we selected for Raman microscopy scanning. Accordingly, in Fig. 4 (top) we present a typical Raman spectrum sampled at the surface of the conidiophore: see the red mark in the middle panel in Fig. 3. Two intense bands at 1350 and 1600 cm^−1^ and relatively weak Raman peaks at 1444 and 1700 cm^−1^ are observed. It is important to note that we do not observe Raman peak at about 790 cm ^−1^. In the higher wavenumber spectral range, the intense peak at 2930 cm^−1^ is accompanied by a weaker one at 2880 cm^−1^ and a higher frequency broad shoulder at 3000 cm^−1^. We note that the spectrum does not show Raman peaks in the spectral range 1090–1150 cm^−1^, typical for polysaccharides in several different fungi and plant cells.^13,19,20,37^ This observation is interesting for two reasons. First, the results of early studies of chemical composition suggest that chitin and glucans should dominate in hyphal walls of *Botrytis cinerea*.^45,46^ Second, the spectrum we report here resembles both, early Raman spectra measured on *Cladosporium* sp. hyphae^22^ and recent spectra from the filaments of *Colletotrichum* coccodes.^23^ In the following section we suggest spectral assignments based on quantum chemical calculations and discuss Raman microscopy as a tool to address cellular physiology. To assign the observed resonances we computed Raman activities of the model molecular systems selected according to the results of prior analytical chemistry studies. Specifically, material studies suggested that *Botrytis cinerea* filaments should include chitin^45^ and glycoproteins as the main structural components with any lipid present being at significantly lower levels.^45–47^ Confocal microscopy support for the presence of chitin in our samples as shown in Fig. 5.

As we used a 532 nm wavelength for excitation in our experiment, we expected glycoproteins and polysaccharides to provide non-resonant Raman signals. Additionally, based on prior studies which indicated that depending on maturity, *B. cinerea* is expected to show deposition of DHN-based melanin,^34^ we considered a DHN chromophore as an early melanin precursor, its more structurally complex dimer DHN_2_, and a highly condensed PQN derivative: Fig. 2. Considering that the excitation wavelength is close to the electronic resonances of melanin chromophores, we have to account for possible pre-resonant contributions that may affect Raman intensities for these chromophores when excited at 532 nm. Specifically, we quantify Raman enhancement in dependence on detuning from HOMO–LUMO transition wavelengths specific to the considered chromophores: Fig. S2 and S3 in the ESI† specify DFT predictions for Raman intensity enhancement for different DHN chromophores to vary from 100 to 50 000. The computed enhancements support our expectation to observe pre-resonant Raman from DHN chromophores in the filaments.

**Fig. 6:**
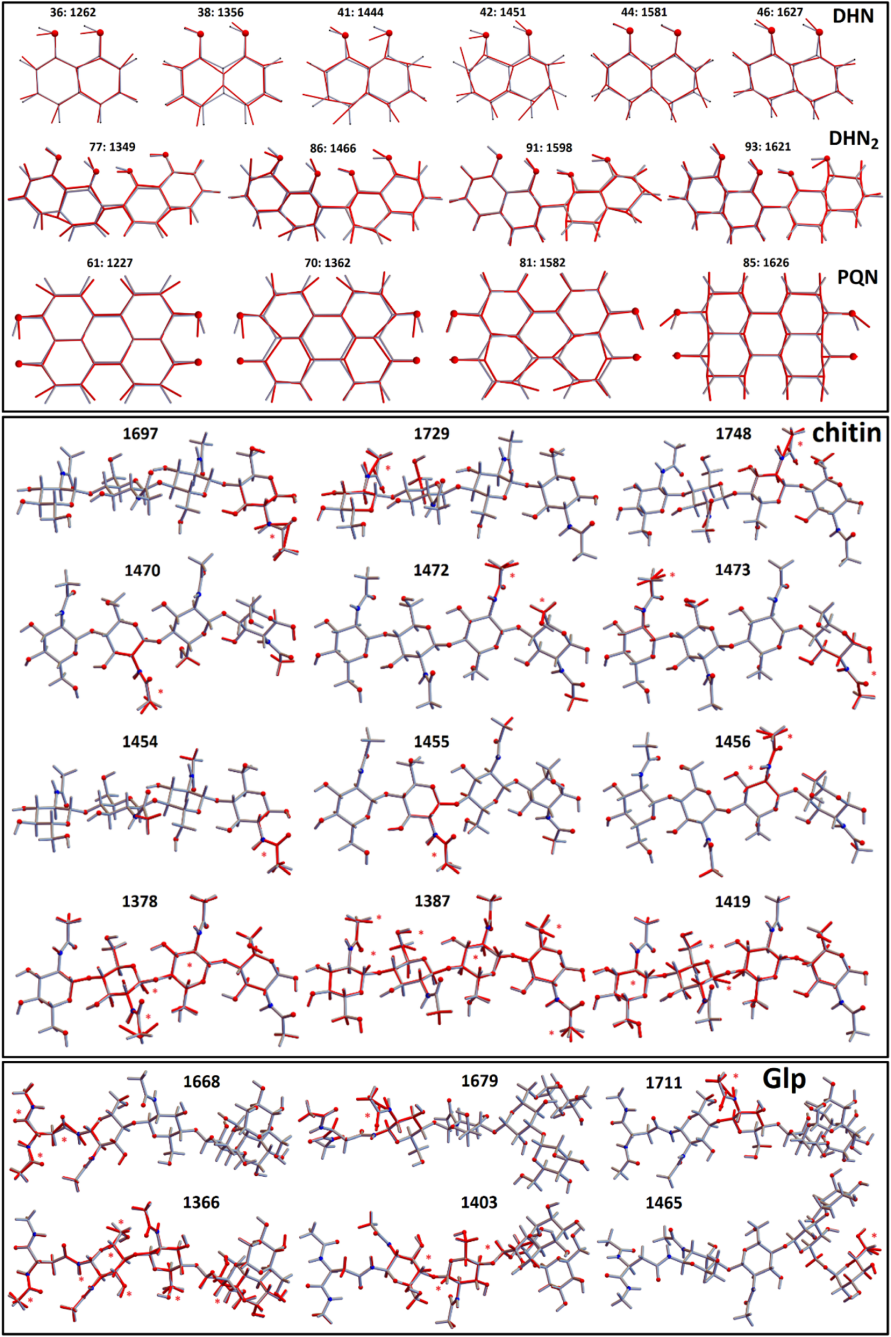
Model molecular systems: 1,8-dihydroxynaphthalene (DHN), DHN-2-2′-dimer (DHN_2_), perylenequinone (PQN), glycopeptide (Glp), α-chitin segment; and displacements of representative normal modes. Red stars indicate largest displacements. Numbers are Raman wavenumbers computed using DFT and using scaling factor 0.97.

In Fig. 4 we present Raman spectra computed for the model molecular systems, as shown in Fig. 2. Furthermore, in Fig. 6 we describe graphically computed atomic displacements along the normal modes. Linking experimental Raman spectra with theoretical predictions, spectra of DHN and DHN_2_ can be explained well in the spectral range between 800 and 1620 cm^−1^. Further, computational DFT studies predict that, when excited at 532 nm, Raman resonances of DHN based molecular systems should demonstrate significant intensity enhancement due to pre-resonance of excitation wavelength with electronic transition. Accordingly, we suggest the following assignments. Raman band at 1350 cm^−1^ should be mainly due to C_4a_C_8a_ stretching associated with C_4a(5a)_C_4(5)_H and C_8_OH bending of the DHN mode 38; and the analogous DHN_2_ mode 77. Raman peak at 1450 cm^−1^ may be described by (a) C_4_C_4a_–C_8_C_8a_ symmetric stretching of the DHN mode 41, (b) C_1_C_8a_–C_8a_C_8_ + C_1_C_8a_–C_8a_C_8_ antisymmetric stretching of the DHN mode 42, and (c) C_1_C_8a_–C_7_C_2'_–C_8'_C_8a'_ symmetric stretching of the DHN_2_ mode 86. Raman band at 1600 cm^−1^ should be mainly due to (a) DHN mode 44 stretching of the aromatic ring along the vertical dimension: C_2_C_3_–C_4a_C_8a_–C_6_C_7_, (b) not shown in Fig. 6 but similar stretching mode 89 at 1567 cm^−1^ along the vertical dimension of one of the aromatic pairs in DHN_2_, (c) DHN_2_ mode 91 accounting symmetric stretching C_3'_C_4'_–C_1'_C_8a'_–C_8_C_8a'_–C_5'_C_6'_, and (d) DHN_2_ mode 93 where the same stretching mode as in the mode 91 but involving both aromatic sets. In all cases the skeletal C–C stretching are associated with CCH bending and, sometimes, with COH bendings as shown in Fig. 6.

Since experimental spectra do not show any obvious Raman activity between 1220 and 1230 cm^−1^, we exclude possible contribution of PQN molecular systems and similar. However, in Fig. 6 we provide normal descriptions as calculated for this case. The presence of highly condensed PQN like molecular systems is expected along the pathway of DHN-melanin synthesis in fungi.^39–41^ The lack of such a molecular component may explain the light-gray coloration of the *Botrytis* filaments explored in this study that include DHN and DHN_2_ like precursors only.

Previously, researchers have tried to assign spectroscopic bands to specific fungal signatures for melanized *Cladosporium* hyphae and spores;^22^ bands at 1330, 1454 and 1590 cm^−1^ were ascribed to CH deformations, CH_2_ and CH_3_ deformations, and aromatic and aliphatic C=C unsaturated stretching and CCH stretching, respectively. In a recent microscopy study,^23^ spectral responses were ascribed to chitin. Our experimental results strongly supported with a theoretical understanding of the transitions involved suggest that all three bands are dominated by the electronically enhanced responses of in-plane aromatic C=C unsaturated stretching admixed with CCH bendings.

DFT predicts that, when excited at 532 nm, Raman spectral signatures of DHN-based molecular systems should demonstrate electronic pre-resonant enhancement, but do not contribute to Raman resonances in the spectral ranges between 1630 and 1750 cm^−1^ and between 2850 and 3050 cm^−1^, hence we considered these spectral windows to be informative mainly on the chitin and glycopeptide structural component.^45–47^ Due to the non-resonant character in respect to the 532 nm excitation wavelength used, we cannot expect responses from these components to be intense. Nonetheless, since the computed CH stretching modes at 2900 cm^−1^ demonstrate 10–20 times stronger activity than any mode at the lower frequency range, between 700 and 1800 cm^−1^, (see Fig. 4), this provides a rationale for how non-resonant behavior of chitin and glycopeptides may provide the observed Raman at *ca.* 3000 cm^−1^, while we did not observe obvious spectral signatures of these structural components at 1100 cm^−1^.

Comparing the computed responses of chitin and glycopeptide model systems with experiment in the spectral range from 2850 to 3050 cm^−1^, we suggest that while the Raman of glycopeptides should contribute to the sharp peak at 2930 cm^−1^ mainly, chitin and other polysaccharides would contribute in the full range of the band thus providing for the broadening of the peak and the spectral shoulders. This interpretation is reasonable when we compare our data to a recent study of pure chitin (α, β and γ) sheets,^48^ where the main Raman peak in this spectral range is blue shifted, at 2940 cm^−1^, though its relative intensity (comparing to other CH modes) is not as dominant as the main feature we observe in our experimental studies. Hyphal walls should present a wider diversity of local sites (for CH moieties) than in a pure chitin sheet.^49^ Therefore, the computed broad distribution of Raman activities of localized CH modes of the relatively short (well-extended in space) model chitin segment may be considered as a good predictor with the caveat, that the wide range of chemical sites, for CH resonances under spectral homogeneity,^48^ would provide the spectrally broad band, extended to the higher frequency range. In comparison, for the glycopeptide, theory predicts that, the relative spectral narrowing and the larger intensity of the CH stretching band at 2950 cm^−1^ may be ascribed to the more compact and folded character of the glycopeptide side-group. In result, stretching of the nearby –CH_2_–OH and CH_3_ moieties, demonstrate better couplings to provide delocalizations. Frequencies of such modes are more red-shifted, and their intensities are larger. For chitin, theory predicts analogous but more local and less intense CH stretching modes to dominate at the higher wavenumber range: from 2970 to 3100 cm^−1^. To further justify our findings for the presence of chitin, and to investigate cell wall formation as well as the structure of the hyphae, calcofluor staining was done. This fluorochrome binds nonspecifically to chitin segments in fungi. The blue fluorescence images show the presence of chitin in the cell wall of *B. cinerea* in the early stage of growth (48 h). The hyphae seem to have true structures with septum formation^50^ (Fig. 5A–C). Hence the presence of chitin as identified by Raman is further supported.

Graphical images in Fig. 6 describe theory predictions of the nature of the Amide I normal modes of chitin and a glycopeptide model system. The Amide I modes of chitin tend to express at higher wavenumber range (1700–1750 cm^−1^) due to their localized nature. In contrast, the Amide I modes of an exemplar glycopeptide demonstrate a diversity of Amide I modes that involve protein backbone amide units as well as contributions of the amide linker to the glycosyl structural component. Such Amide I modes tend to express at the lower frequency side of the spectral range from 1650–1710 cm^−1^. At the same time, theory predicts that localized Amide I modes of the amide linker may have resonances at the higher frequency side of the spectral range from 1650–1710 cm^−1^. Beside the Amide I modes, in Fig. 6 we present graphical presentations for typical CH_2_/CH_3_ and CH bending modes, which determine Raman activities in the 1450–1470 cm^−1^ and 1320–1390 cm^−1^ spectral ranges, respectively. According to our experimental conditions and theory predictions, these Raman responses are most likely at background levels because pre-resonant responses of DHN-based molecular components dominate the experimental response in the indicated spectral range. In conclusion, here, we update the previous description of Raman responses of a plant fungal pathogen using functional group assignment^22^ with a molecular based description. Here, it is important to note that in the spectral range studied we may additionally have expected contributions from nucleic acids. However, as we did not observe the characteristic DNA Raman peak at about 790 cm^−1^, we may deduce that, if nucleic acids are present, this is below the sensitivity of detection. Since analogous experimental results are reported for other plant pathogens,^22,23^ we ascribe this to dominance of pre-resonant Raman responses from DHN-melanin chromophores. At the same time, we cannot rule out a decay of the organelle or an evacuation *via* openings in septa, which may happen after completion of conidiophore differentiation.

**Fig. 7:**
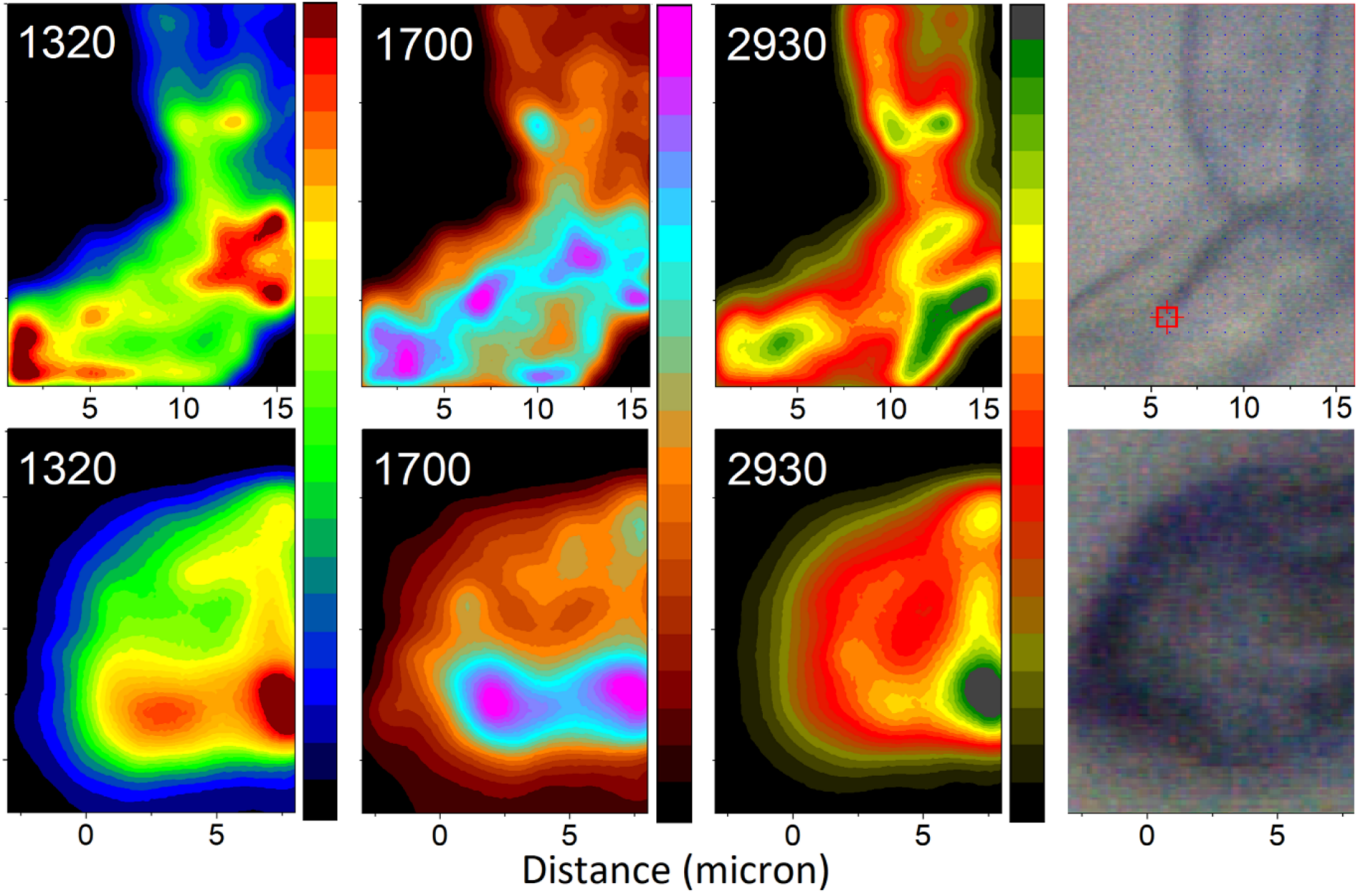
Raman microscopy images of a selected hyphal region (top) and a spore (bottom) reconstructed using frequencies (wavenumber) of Raman peaks as indicated. Images reconstructed at 1320 cm^−1^ largely represent contributions from melanin precursors: modes 38 and 77 of DHN and DHN_2_, as in Fig. 6. Images reconstructed at 1700 cm^−1^ represent contributions largely from glycopeptide/chitin components: resonances of Amide I and of carboxyl groups and, to lesser extent, of chitin Amide I of acetyl amine side groups. Images reconstructed at 2930 cm^−1^ present contributions from C–H stretching of chitin and glycopeptide. The microscopy photos on the right-hand side, as per Fig. 3, image the areas explored by Raman microscopy. To contrast differences in chemical distributions we adopt different color schemes as indicated in the figure. In the ESI† file, we present the images using the same color scheme, and we provide the color bars with numerical scales.

Having assigned the spectral responses, we may explore distributions of molecular components in the organelles under study: see Fig. 7. Specifically, we anticipate the images reconstructed using Raman activities at 1320 cm^−1^ to characterize distribution of DHN-based chromophores. Furthermore, the Raman map computed for intensities in the spectral region at 1700 cm^−1^ should characterise the presence of glycopeptides (resonances of Amide I and of carboxyl groups), and, to lesser extent, of chitin (resonances of Amide I of acetyl amine side groups). Finally, the Raman image reconstructed using intensities at 2930 cm^−1^ describes the distribution of CH stretching of chitin and glycopeptides. To contrast differences in chemical distributions we adopt different color schemes as indicated in the figure. In the ESI† file, we present these images using the same color scheme, and we provide color bars with numerical scales to facilitate further comparison. The suggested assignment is according to the results of theoretical spectral analysis and chemical studies on the major components present in Ascomycetes structures.^51^ Following this, next, we are ready to relate the chemical distributions in space to the biology of the selected organelles.

Aside from providing a pathway for transport of materials upon spore formation, the conidiophore stem principally plays a structural role to provide both, stability, and flexibility. For this purpose, the conidiophore is biochemically active until reproductive dispersal. In contrast, function of a spore is unequivocal – stability of storage. From this perspective, it is important that the images reconstructed at 2930 cm^−1^ describe signatures characteristic to the structural-constituent mainframe (scaffold) of the organelles being studied. The higher intensity (yellow to green coloring) areas in the images indicate where CH stretches are more parallel to the field vector of the excitation field. These are the regions, where the surface experiences a tilt: either as we sample at a side of the organelle or at a side of a concave region, or in a junction region, where surfaces of neighbouring filaments bend towards one another. The dominant intensity spot (dark green) suggests that in our scan, the field (of 532 nm wavelength) does not refract into the sample the internal content as was reported recently for *Aspergillus nidulans* spores.^18^ Instead, we sample mainly Raman signals reflected from the surface of the spore. One may consider that melanin depositions, evolutionarily selected for photo-protection, may aid effective reflection to protect internal cellular contents.

Now, it is interesting to account how the distributions of intensities of other Raman activities relate in space with such at 2930 cm^−1^. In the case of the spore, the distribution of Raman activities of DHN-based chromophores at 1320 cm^−1^ largely coincides with the distribution of Raman activities of CH modes at 2930 cm^−1^. We consider the uniform deposition of such pigments to provide optimal photoprotection of the content. In this respect, we may suggest that electronic properties of deposited DHN pigments may help provide the observed highly reflective character of the surface of the *Botrytis cinerea* spore. If such is the case, this may be considered as an evolutionary benefit to serve the plant pathogen as it thrives in geographical regions of high light exposure.

The distribution of Raman activities of DHN-based chromophores at 1320 cm^−1^ is not the same in the case of the hyphal region of the conidiophore. There, we observe some preference in deposition of DHN-based pigments in the region of branch junctions. The deposition suggests that at the site we may detect a higher presence of hydrophobins,^52^ and/or signatures of actin and transport proteins necessary during development of spores,^53^ where connection to the ‘parent’ will cease on sporulation. To understand this better we explore the distributions of Raman activities in the spectral region at 1700 cm^−1^, which we associate with Amide I and carboxyl groups of glycopeptides, as well as Amide I modes of chitin, which are molecular types involved in defining structural elements. In this respect, in the spore map at 1700 cm^−1^, it is interesting to observe the intense spot (the left magenta region) in the spatial region, where the conidiophore was attached to the spore.

For the same Raman peak at 1700 cm^−1^, the conidiophore map presents a pattern, which tends to correlate better with the image characteristic to DHN-based pigments. The image suggests that protein distribution tends to co-align with the branching folds in the bright field image (at the right side in Fig. 7). The higher complexity of the high Raman intensity (than for other cases) may suggest that the conidiophore image at 1700 cm^−1^ includes signatures of molecular components involved in melanin biosynthesis,^54^ of glycoproteins and chitin structural components, and of proteins that support filament transport.^55^

Here it is important to note that the spectra we report here for *Botrytis cinerea* are reminiscent of the Raman spectral features measured on *Cladosporium* sp. hyphae,^22^ and spectra detected in the filaments of *Colletotrichum coccodes*^23^ and the spectrum of a particular *Aspergillus fumigatus* ayg1Δ strain.^21^ Interestingly *Botrytis*, *Cladosporium*, *Colletotrichum* belonging to the classes of *Leotiomycetes*, *Dothideomycetes*, and *Sordariomycetes* respectively, under the common division of Ascomycota, and are all important plant pathogens. The similarity of Raman responses may reflect similar structural features (here, at the molecular level) by adapting to a similar environment. In this respect, assignments of spectral responses (at the molecular level) may be important for better understanding how evolutionary genetic diversities, nonetheless, provides the observed evolutionary convergence at the level of the secondary naphthalene-based metabolites deposited as UV protecting pigments of maturity. Finally, here, considering that plant pathogenic fungi present an advanced group, we anticipate the diversity of melanin biosynthesis reported for saprotrophic species^56^ to reflect biochemistry characteristic of the ancestor.

## 4. Conclusions

To discuss the biology of *Botrytis cinerea* upon maturation, we employ Raman and fluorescence confocal microscopy to map the chemical composition of conidiophore and hyphae. To understand Raman resonances, we use quantum chemistry to compute Raman tensors specific to model glycopeptide and hydrocarbon molecular systems expected in such organisms. We update the previous description of Raman responses of plant pathogenic fungi using functional group assignment^22^ with a molecular based description of biochemical diversity. Specifically, we ascribe three Raman bands at 1350, 1450 and at 1600 cm^−1^ to modes of naphthalene-based pigments, as anticipated along the fungal melanin synthesis pathway.^39–41^ Furthermore, we suggest that the spectral windows between 1630 and 1750 cm^−1^ and between 2850 and 3050 cm^−1^ are informative on Raman responses of chitin and glycopeptide constituent components.^45–47^ Using the assignments, we compare microscopy images reconstructed for selected Raman activities to discuss differences in distributions of structural components, photo-protective secondary naphthalene-based pigments and proteins in conidiophore and hyphae. In accord with literature data for other fungal classes, we hypothesize that the reported naphthalene-based pigments may present an example of convergent evolution expressed at the level of secondary metabolites specific to plant pathogens.^22,23^

## Author contributions

Victor V. Volkov: investigation, methodology, software, analysis, writing. Ayesha Sadaf: investigation, writing. Carole Perry: funding acquisition, theoretical studies, methodology, writing.

## Conflicts of interest

The authors declare no conflicts.

## Supplementary Material

RA-013-D2RA06439A-s001
