# Supplementary material for: Raman microscopy tracks maturity of melanin intermediates in Botrytis cinerea, a plant pathogen
Source: RSC Adv. 2023 Jan 6;13(2):1381–91. doi: 10.1039/d2ra06439a (PMC9817083; doi:10.1039/d2ra06439a)
Supplement: RA-013-D2RA06439A-s001 [file RA-013-D2RA06439A-s001.pdf]

This journal is © The Royal Society of Chemistry 2022

Supplementary Materials

Raman microscopy tracks maturity of melanin intermediates in *Botrytis cinerea, a plant pathogen*

*Victor V. Volkov, Ayesha Sadaf and Carole C. Perry**

Interdisciplinary Biomedical Research Centre, School of Science and Technology, Nottingham Trent University, Nottingham, United Kingdom

Corresponding Author: Professor Carole C. Perry

Interdisciplinary Biomedical Research Centre, School of Science and Technology, Nottingham Trent University, Nottingham, United Kingdom NG11 8NS

+44 115 8486695

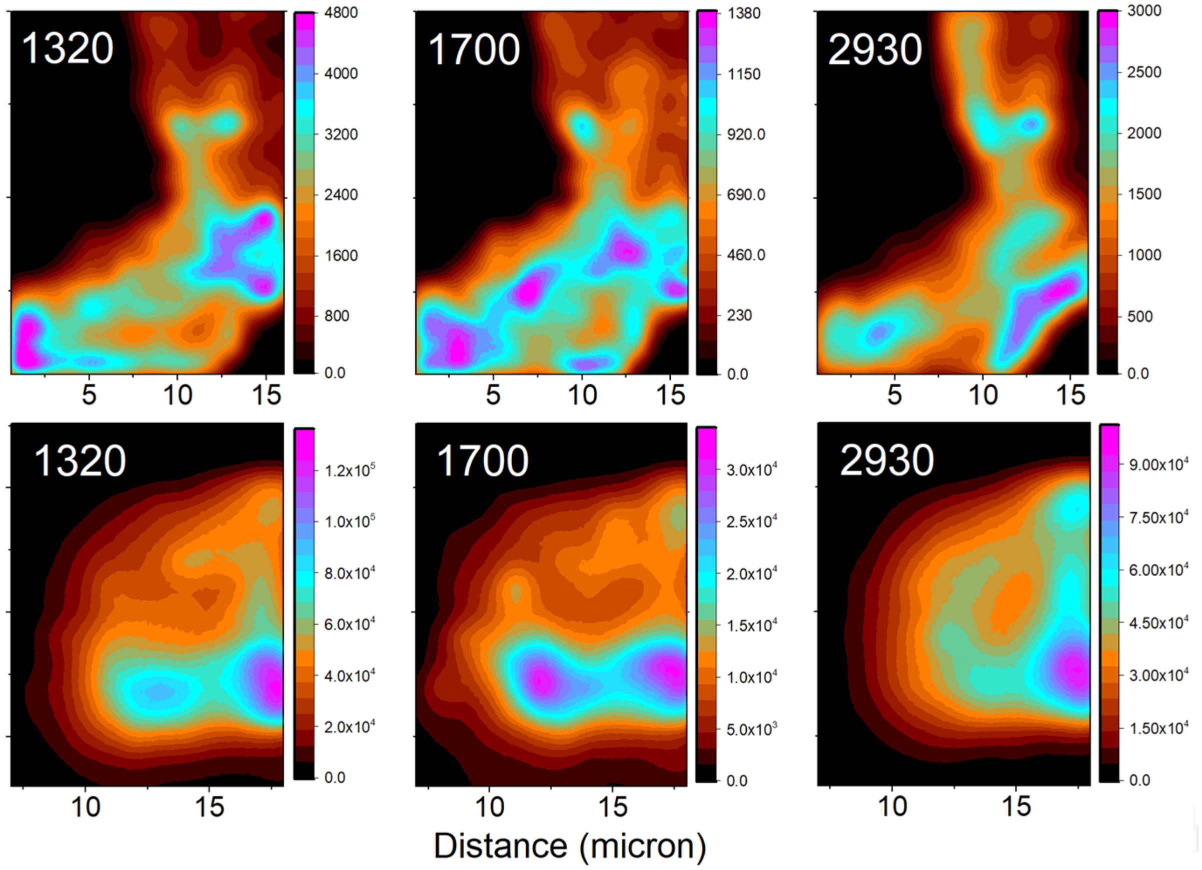

Figure S1. *Raman microscopy images of a selected hyphal region (top) and a spore (bottom) reconstructed using frequencies (wavenumber) of Raman peaks, as indicated. The images present the same data as in Figure 7 in the main text but under the same color scheme and with the color bars numerically scaled.*

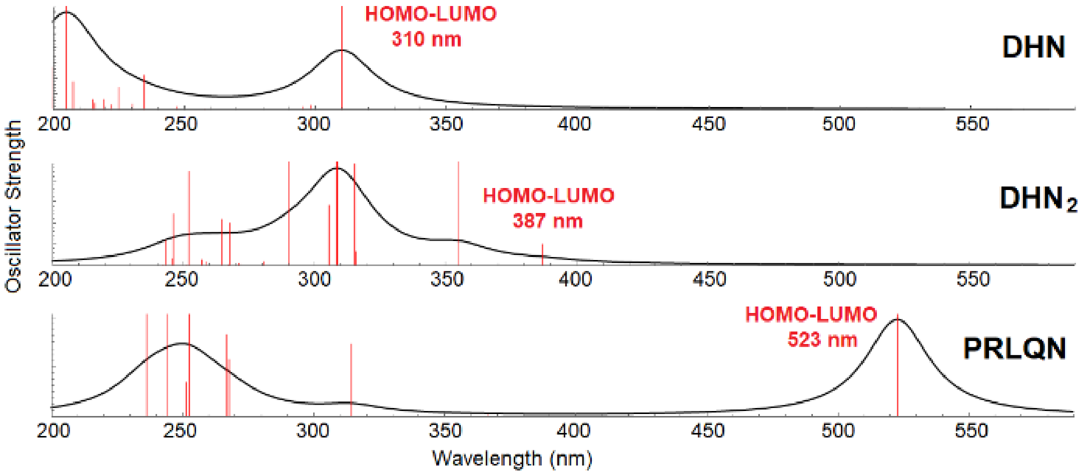

Figure S2. *UV-VIS spectra computed for* 1,8-dihydroxynaphthalene (DHN), DHN-2-2'-dimer (DHN_2) and perylenequinone (PRLQN) *chromophores with indication of highest occupied molecular orbital (HOMO) and lowest unoccupied molecular orbital (LUMO). The proximity of the excitation wavelength to the indicated transitions may provide an enhancement of Raman intensity.*

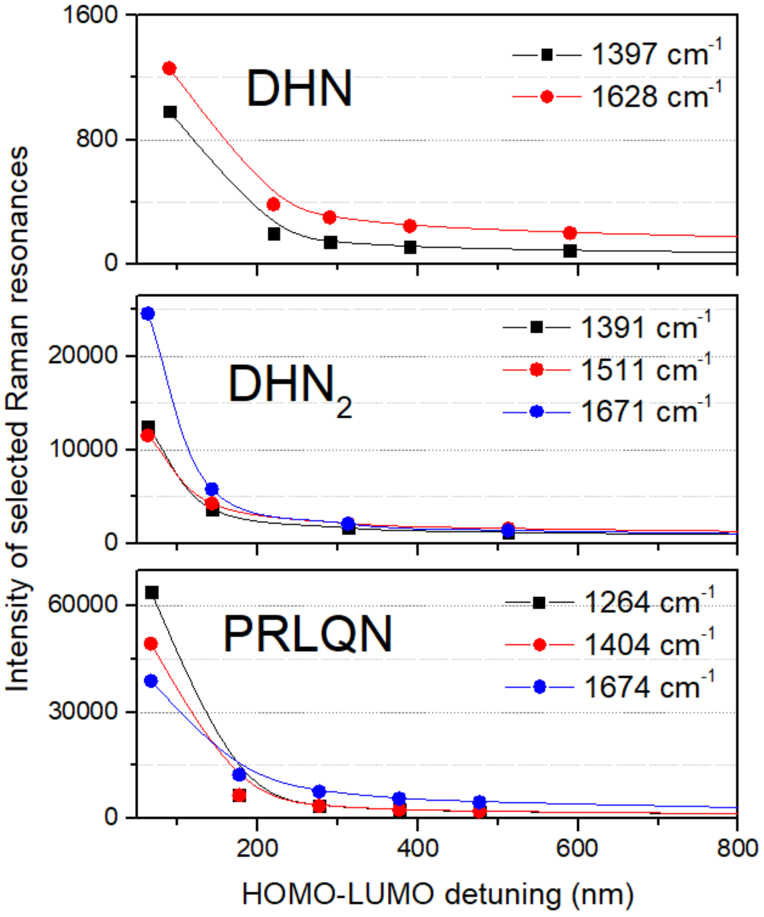

Figure S3. *Raman intensity of the normal modes at the frequencies as indicated in dependence on the detuning of the incident light wavelength (for the electromagnetic field perturbation upon DFT studies) in respect to the wavelength of HOMO-LUMO transitions. The nonlinear enhancement of the Raman intensities when the excitation wavelength becomes close to HOMO-LUMO transition suggests the importance of accounting for this when computing Raman spectra for such chromophores to compare with experiment.*
